# Strategic priorities for accelerating action to reduce the burden of snakebite

**DOI:** 10.1371/journal.pgph.0002866

**Published:** 2024-02-05

**Authors:** Soumyadeep Bhaumik, Abdulrazaq G. Habib, Vishal Santra

**Affiliations:** 1 Injury Division, George Institute for Global Health, New Delhi, India; 2 Meta-research and Evidence Synthesis Unit, Health Systems Science, George Institute for Global Health, Sydney, Australia; 3 Infectious Disease and Tropical Medicine Unit, Department of Medicine, College of Health Science, Bayero University, Kano, Nigeria; 4 Society for Nature Conservation, Research and Community Engagement (CONCERN), Nalikul, Hooghly, West Bengal, India; PLOS: Public Library of Science, UNITED STATES

Snakebite is a public health problem in many low-and middle-income nations. It is estimated that every year there are 5.4 million snakebites leading to 81,000 to 138,000 deaths, mostly in South Asia, Central, Eastern and Western Africa, and to a lesser extent in South America [1]. Snakebite predominantly affects rural and Indigenous communities, children, young adults involved in agricultural activities, and those from lower socio-economic status. However, the recognition of its burden at the global stage is recent. The World Health Organization (WHO) added snakebite to its list of neglected tropical diseases (NTDs) in 2017. Subsequently in 2019, WHO set an ambitious target of halving the burden of snakebite by 2030 and identified four pillars of action: ensuring safe and effective treatments; empowering and engaging communities; strengthening health systems; and increasing partnerships, coordination, and resources [1, 2]. The strategy envisages full global roll-out in 2025, which is just a year away–thus providing an opportunity to take stock, critically reflect, and identify strategic priorities which can accelerate actions to reduce the global burden of snakebite.

## Better epidemiology for better accountability

A key challenge for any snakebite prevention and control initiative is the current lack of epidemiological data on the scale of the problem. Population-level estimates are almost absent in every high-burden nation [3]. In terms of helping guide progress towards snakebite reduction goals, this is akin to “hitting targets while being blind-folded.” It might even be counterproductive.

Robust epidemiological data is not only important for policy, programming, and research, but it is also crucial for WHO to monitor its global targets. Without baseline data, it is impossible to ensure accountability towards the 50% reduction target progress [4]. With global roll-out only a year away [2], it is critical to develop tools for epidemiological studies and establish funding schemes for country-level actors in South Asia and Africa to conduct robust population-based surveys on burden of snakebite. It is also crucial to understand the localised nature of snakebites [3]. This necessitates sub-national surveys in high-burden nations, using innovative methods such as the community knowledge approach, which was developed first in Bangladesh and subsequently tested elsewhere, and for various conditions [5, 6].

## Making snakebite prevention evidence-informed and transdisciplinary

Snakebite is essentially a function of human-environment-snake conflict [4, 7]. As such, only understanding human risk factors is not sufficient for snakebite prevention. Snakebite prevention programs need to be evidence-informed, including using geo-spatial information on snake habitats and research on snake behaviours. This data, if appropriately prepared and analysed, can provide information for better design of strategies for mitigating human-environment-snake conflict [7]. Snakes also play a vital role in maintaining food chains in agricultural areas, crucial for ecosystems and economic livelihood alike. Snakebite prevention programs should be co-designed with communities and teams with expertise in public health, conservation science, ecology, agricultural science, anthropology, and herpetology [8, 9].

The lack of funders on community-based interventions and health systems strengthening for snakebite is also an issue of pressing concern. Designing and funding evidence-informed community-based interventions and promoting optimum development, deployment, and utilization of conventional, effective snake antivenom should be priority over lop-sided funding being made available for research on next-generation immune-recombinant antivenom [4].

## Future-proofing affordability and access to safe, effective treatments

The WHO has made tremendous progress on the pillar for ensuring safe, effective treatments. In many countries of Africa, snake antivenoms (SAV) products that are not suitable for use are still being marketed, which leads to poor clinical outcomes and undermining market confidence [10, 11]. Many African regulatory agencies, drug control laboratories and health authorities lack the resources and technical capacity to adequately regulate and control the safety, effectiveness, and quality of SAVs marketed in African countries [11]. To facilitate countries’ purchase of quality SAVs, WHO has developed a pre-qualification framework that specifies minimum manufacturing benchmarks and Target Product Profiles (TPP). The work of WHO will support countries to assess and register SAV products, approve marketing and evaluate compliance with Good Manufacturing Practices (GMP) and validation of SAV quality.

However, these are not enough in Africa. Unlike Asia and South America, which have many SAV manufacturers, African nations have very few local producers and depend on imported commercial products from other parts of the world [11]. Thus, funding for local development of SAV should be a serious priority. To promote sustainability and viability, in terms of SAV market, countries with similar snake fauna causing envenoming might pool manufacturing facilities to supply their subregion. Public-public partnerships, such as successfully implemented in Costa Rica and other South American countries [12], should be explored to keep SAV costs affordable. In addition to the financial investments needed for local SAV production, there is the technical complexity of establishing and sustaining production locally. Governments might also consider the option of shared quality control and manufacturing entities, with serpentarium to collect high quality venoms being set up at the country-level. This approach for gradual consolidation of local capacities should be led by African stakeholders and have trans-national implementation frameworks. Development of such frameworks will require proactive engagement of the African Union and WHO.

The influx of funding (primarily from Wellcome Trust) after the prioritisation of snakebite by WHO has led to flurry of research on development of next generation SAVs, Small Molecule Therapies (SMT), repurposed drugs and snakebite diagnostics [13]. While these would not by themselves achieve the 2030 targets, WHO and global funders should ensure that intellectual property of new therapeutics and diagnostics should be owned by public or academic entities in endemic nations, and not entities in non-endemic nations.

Snakebite is a largely disease of the poor and low- and middle-income countries. The 2019 WHO strategy for prevention and control of snakebite is currently intellectual property blind [4], and the world learned during the roll out of COVID vaccines that such an approach can prevent access to life-saving therapeutics. A mid-term revision by WHO to ensure intellectual property rights for SAVs and all new products being marketed is essential. Lessons learnt from campaigns like “People Over Profit,” for improving access to tuberculosis drugs and “MSF Access” might be harnessed for this purpose. Turning a blind eye to access to SAV issues is an injustice [14]. Taking a pro-poor position on intellectual property would prevent endemic countries (mostly low and middle income) being forced to buy SAV and related therapies from foreign entities at prohibitive costs in the future.

## Towards a future where snakebite is no longer a public health problem

Historically, there have been crescendos in the policy prioritisation of snakebite on global health agendas [14]. The historic neglect and lack of funding for snakebite has often been frustrating and resulted in a preventable loss of life. With the current renewed attention by the WHO and other global actors, this is a pivotal moment to ensure sustainable, effective snakebite prevention programs are firmly established in high-burden countries. We cannot miss this opportunity to ensure safe, equitable access to these lifesaving programs where they are needed the most.
